# Efficacy of a Ceftazidime-loaded Nanofiber Insert in Treating *Pseudomonas aeruginosa*-induced Corneal Ulcers: An Animal Model

**DOI:** 10.18502/jovr.v20.15595

**Published:** 2025-10-30

**Authors:** Shahla Mirzaeei, Mojtaba Eidizadeh, Abbas Khosravi, Masood Bagheri, Shiva Taghe

**Affiliations:** ^1^Pharmaceutical Sciences Research Center, Health Institute, Kermanshah University of Medical Sciences, Kermanshah, Iran; ^2^Pharmaceutical Sciences Research Center, Rahesh Daru Novine, Kermanshah, Iran; ^3^Nano Drug Delivery Research Center, Health Technology Institute, Kermanshah University of Medical Sciences, Kermanshah, Iran; ^4^Clinical Research Development Center, Imam Khomeini and Mohammad Kermanshahi and Farabi Hospitals, Kermanshah University of Medical Sciences, Kermanshah, Iran; ^5^Department of Ophthalmology, Imam Khomeini Eye Center, Kermanshah University of Medical Sciences, Kermanshah, Iran

**Keywords:** Animal Model, Ceftazidime, Corneal Ulcer, Drug Delivery, Nanofiber Insert

## Abstract

**Purpose:**

This study aimed to determine the efficacy of ceftazidime-containing nanofibers in treating corneal ulcers induced by the bacterium *Pseudomonas aeruginosa* in an animal model.

**Methods:**

This animal-assisted intervention involved 12 adult male New Zealand rabbits, each weighing between 3.5 and 4 kg. The animals were randomly assigned to two groups of six: an intervention group that received a ceftazidime nanofiber insert treatment and a control group that received no treatment. In the intervention group, the right eye was used as a test sample for ulcer induction and ceftazidime-loaded nanofiber examination, while the left eye served as a control to observe any inflammatory or irritating symptoms caused by the nanofiber in the absence of the active pharmaceutical compound. Examinations were performed daily, with slit lamp images taken on days 2, 4, 6, 9, 12, and 15. Clinical responses were recorded and graded according to a clinical examination table.

**Results:**

Prior to the intervention, both groups exhibited a similar severity of corneal ulcers. After 48 hours, four of the six rabbits in the intervention group were positive for *Pseudomonas aeruginosa* in their cultures, and the remaining two tested negative. Meanwhile, in the control group, three rabbits had positive cultures and three had negative cultures. After 96 hours from the onset of the treatment and the application of the ceftazidime nanofiber insert, three rabbits that initially presented with positive cultures exhibited negative cultures in the subsequent examinations; however, one rabbit still had positive smear and culture results. On day 9, the intervention group showed complete disappearance of infiltration and epithelial damage. However, the rabbit eyes in the control group demonstrated increased signs of involvement on days 6 and 9 compared to the previous examinations. Furthermore, the clinical results indicated a significant difference in the mean corneal ulcer scores between the two groups (*P* < 0.001).

**Conclusion:**

Given the observed effectiveness of the developed nanofiber in treating corneal ulcers induced by* P. aeruginosa*, this nanodrug delivery system has the potential to serve as a viable option for ocular drug delivery.

##  INTRODUCTION

Corneal ulcers, a potential vision-threatening emergency and a common cause of visual impairment, are mainly caused by bacterial pathogens such as *Staphylococcus aureus*, *coagulase-negative staphylococci*, and *Pseudomonas aeruginosa*.^[1,2]^ Even with immediate treatment, patients with corneal ulcers may still experience significant complications. Therefore, preventive measures, early diagnosis, and timely treatment are crucial to avoiding long-term vision loss.^[3]^

The main drawback of ocular drug delivery is its relatively low efficiency, as the unique physiology and anatomy of the eye surface can result in limited drug bioavailability.^[4]^ Corneal ulcers are typically treated with topical medications, such as eye drops, which require frequent application throughout the day.^[5]^ However, several issues may arise with the use of eye drops, including inadequate intraocular drug concentration throughout the day, toxicity from high drug concentrations during the first hour of use, concentration fluctuations, side effects caused by preservatives, and patients' noncompliance with the treatment regimen.^[6,7]^ Therefore, an efficient delivery system is required to control drug release and decrease dosing frequency.^[8,9]^ Accordingly, different drug administration methods have been developed to maintain a steady dosage of medication, aiming to achieve consistent therapeutic effects while minimizing side effects. One such method is the use of nanofiber inserts.^[10]^

**Figure 1:**
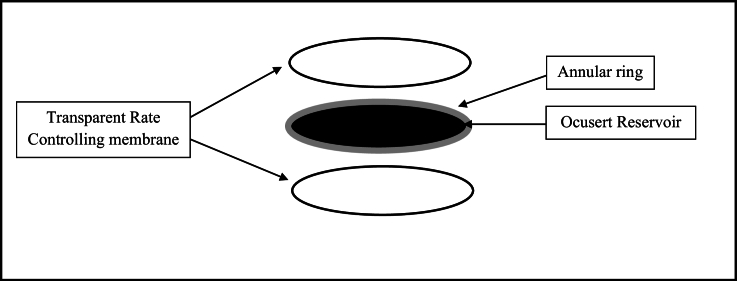
Schematic image of an ophthalmic insert. These systems comprise three components, including a central drug reservoir, a rate-controlling membrane, and an outer annular ring.

This innovative system comprises a drug reservoir and a rate-controlling membrane, which utilizes a variety of polymers to regulate drug delivery [Figure 1].^[11,12]^

**Figure 2:**
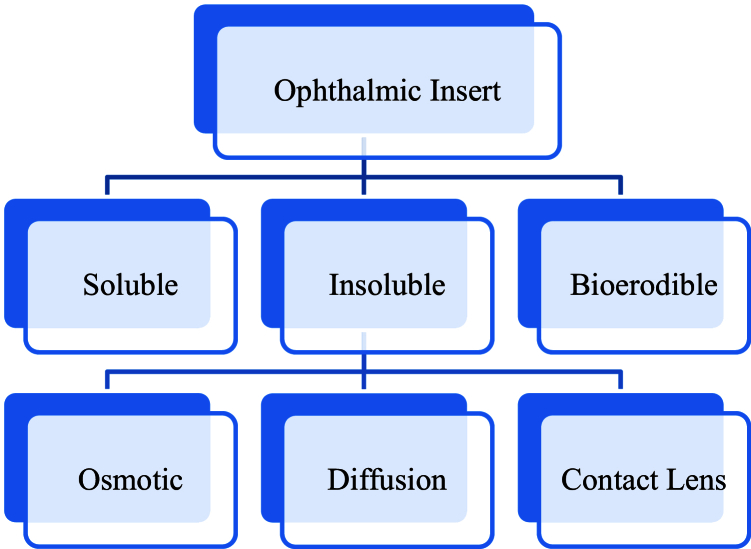
The Ocusert systems can be classified as either soluble, insoluble, or bioerodible. The insoluble inserts are further categorized into three types: Diffusion inserts, osmotic inserts, and soft contact lenses.

These systems can be classified as soluble, insoluble, or bioerodible [Figure 2].^[13]^ Soluble inserts release the drug through the interaction between the device's polymer matrix and the tear film, and their crucial advantage is solubility, which eliminates the need for removal. While insoluble inserts may provide a more stable drug release rate than soluble types, they require removal. These insoluble inserts are further categorized into diffusion inserts, osmotic inserts, and soft contact lenses. Ophthalmic inserts offer the benefits of prolonged and consistent drug delivery, which can enhance patient adherence to the treatment regimen.^[14]^ However, they may cause discomfort, such as a foreign body sensation, which can lead to increased tear production. This discomfort can subsequently decrease drug concentration, potentially reducing the insert's performance and the overall effectiveness of treatment.^[15]^

This study is the first to investigate the efficacy of ceftazidime-containing nanofibers in treating *P. aeruginosa*-induced corneal ulcers in an animal model.

##  METHODS

This animal intervention study involved 12 male New Zealand rabbits of the same age group, each weighing between 3.5 and 4 kg. The rabbits were sourced from the Department of Reproduction and Maintenance of Laboratory Animals at the Pasteur Institute of Iran. They were housed in specialized cages and maintained under identical nutritional and environmental conditions. All phases of this study were carried out at the Faculty of Pharmacy, Kermanshah University of Medical Sciences, Kermanshah, Iran. The study protocol was designed following the guidelines of the International Convention for the Protection of Animals^[16]^ and received approval from the Laboratory Animals Ethics Committee (IR.KUMS.AEC.1401.059).

**Figure 3:**
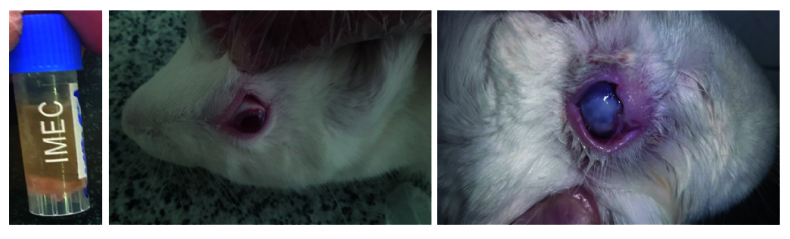
Lipophilized *Pseudomonas aeruginosa* strain ATCC 27853, manufactured by Bahar Afshan Company (Tehran, Iran), which was used to induce corneal ulceration according to the American Type Culture Collection (ATCC; left figure). Rabbit eye irritation test, examining nanofibers in terms of causing irritation symptoms after 72 hours in the healthy left eyes of the rabbit (middle figure) and severe-induced ulcer with abundant mucopurulent secretions in the right eye (right figure).

For the experiments, the animal was first physically restrained with an intramuscular injection of lidocaine and ketamine, dosed at 8 and 100 mg/kg, respectively. Subsequently, local anesthesia was administered using 0.5% tetracaine eye drops (Anestocaine, Sina Darou Co., Tehran, Iran). Initially, an attempt was made to induce the ulcer by inoculating the microorganism after removing the corneal epithelium, which was unsuccessful. Next, the epithelial layer of the corneal surface was removed to a size of 1 mm. Subsequently, 5 μ L of the *P. aeruginosa* strain ATCC 27853—an activated microorganism of 0.5 McFarland in normal saline solution—was injected into the stroma. This procedure was performed according to the American Type Culture Collection (ATCC) standard by Bahar Afshan Company (Tehran, Iran) [Figure 3] using a 29-gauge insulin needle behind a slit lamp. The animal was then monitored for 24–48 hours to check for the formation of ulcers.

At the onset of the treatment, the induced wounds in the animals' eyes were categorized according to severity based on the grading table [Table 1]. The size of the corneal epithelial defect (after fluorescein staining) and corneal opacity/infiltration were measured with a caliber, and the mean diameter was calculated. Once an ulcer had formed, culture and smear samples were prepared from the corneal wound to confirm that the ulcer was monopathogenic. Following this, the animals were divided into two groups. The first group (intervention group) consisted of six rabbits. Medicinal inserts were placed into the inferior conjunctival fornix of the rabbits' eyes. In this group, the right eye of each animal was used as a test sample to induce an ulcer and examine the effects of the ceftazidime-loaded nanofiber. The left eye, on the other hand, was used as a control to study the inflammatory and irritating symptoms caused by the nanofiber in the absence of the active pharmaceutical compound. The irritation test was conducted using clinical methods to evaluate symptoms such as eye discomfort, lacrimation, and eye redness. These evaluations were based on the International Organization for Standardization 10993-10:2010(E).^[17]^

We developed nanofibrous inserts that continuously release ceftazidime, an effective antibiotic against gram-negative and gram-positive bacteria, to treat eye infections. These nanofibers were produced through the electrospinning technique using biodegradable materials such as polyvinyl alcohol, polycaprolactone (PCL), and polymethacrylate polymers. The nanofibrous inserts with an average diameter of < 250 nm exhibited adequate mechanical strength for ocular use. The manufacturing process and properties have already been described in detail in a previous study by this research team.^[18]^ Following extensive physicochemical and biological testing, the formulation that proved to be the most effective in our study was selected for further testing in animal studies. In this research, we aimed to utilize a suitable electrospinning polymer technique to regulate the rate of antibiotic release, ensure long-term release, and enhance the retention time of drugs in the eye. Generally, it is crucial to maintain a steady rate of drug release in the eye in a noninvasive manner. Employing the nanofiber method and an appropriate polymer, we designed a pharmaceutical delivery system that delivers the drug over an extended period when administered into the inferior conjunctival fornix. This method ensures a constant drug concentration within the therapeutic range over time. This approach is highly beneficial as it enhances treatment efficiency, minimizes side effects, and improves patient adherence to the treatment regimen.

**Table 1:** Corneal ulcer clinical scoring based on slit lamp examination

**Grade**	**Focus of infection**		
0	No focus of infection		
1	Corneal infiltrate	1.25	Corneal infiltrate limited in the inoculated area
		1.50	Corneal infiltrate ≤ ½ corneal thickness
		1.75	Corneal infiltrate > ½ corneal thickness
2	Corneal ulcer	2.25	Diameter ≤ 3 mm
		2.50	> 3 mm diameter < 5 mm
		2.75	Diameter ≥ 5 mm
3	Hypopyon	3.25	≤ 1/3 AC altitude
		3.50	> 1/3 AC altitude < ½ AC altitude
		3.75	≥ ½ AC altitude
4	Corneal perforation	4.00	
mm, millimeter; AC, anterior chamber			

The second group (controls) consisted of six rabbits. These animals did not receive any therapeutic intervention, allowing for a comparison of the drug's effectiveness in clinical improvement. Subsequently, the animals underwent daily examinations using a slit lamp to monitor the improvement or progression of clinical symptoms. Slit lamp images were acquired on days 2, 4, 6, 9, 12, and 15. The clinical response was then recorded and graded according to the clinical examination table.

The severity of the corneal ulcer was assessed based on the grading scheme reported by Lee et al.^[19]^ Any changes in medical therapy should therefore be based primarily on clinical response. Several parameters are used for monitoring clinical response to antibiotic therapy:

The size (orthogonal diameters of the major and minor axes) of the epithelial defect (assessed by fluorescein staining) and reepithelialization

• Blunting of the perimeter of the stromal infiltrate
• Decreased density of the stromal infiltrate and edema
• Reduction in anterior chamber inflammation and resolution of hypopyon
• Cessation of corneal thinning
• Corneal vascularization

Eventually, corneal healing was defined as the closure of the epithelial defect (assessed by fluorescein staining), decreased stromal infiltrate and edema density, cessation of corneal thinning, and corneal vascularization.

The collected data were entered into SPSS Version 22.0 (IBM Corp., Armonk, NY, USA) for statistical analysis. The clinical scores of corneal ulcers, as quantitative variables, are reported as the mean ± standard deviation. Depending on the Shapiro-Wilk normality test results, either the *t*-test or the Mann–Whitney U test, as its nonparametric equivalent, was used to compare quantitative variables between the two groups. A *P*-value of < 0.05 was considered statistically significant.

##  RESULTS

### Rabbit Eye Irritation Test

In this study, the eye irritation test was conducted on the left eye of the rabbits in the intervention group. For this purpose, the nanofiber inserts prepared without any active pharmaceutical compound were placed in the inferior conjunctival fornix of the rabbits' left eyes [Figure 3]. The eyes of each animal were observed at specific intervals from day 1 to day 6 post-treatment. The examination results of the rabbits did not show any signs of eye irritation, such as blinking or severe tearing. Furthermore, no reactions were observed in the rabbits' eyes, and symptoms such as conjunctival redness, corneal opacity, and eyelid swelling were absent. Therefore, the insert without the pharmaceutical formulation did not cause any eye irritation [Table 2].

**Table 2:** Ocular irritation grading test in the rabbits' eyes following the application of ceftazidime PCL-EUD nanofiber. The results showed that the insert without the pharmaceutical formulation did not cause any eye irritation

**Parameter**	**Day**						
	**2 ${}^{\text{nd}}$ **	**3 ${}^{\text{rd}}$ **	**4 ${}^{\text{th}}$ **	**5 ${}^{\text{th}}$ **	**6 ${}^{\text{th}}$ **	**7 ${}^{\text{th}}$ **	**8 ${}^{\text{th}}$ **
Corneal opacity	0	0	0	0	0	0	0
Pupillary reaction	0	0	0	0	0	0	0
Conjunctival redness	0	0	0	0	0	0	0
Eyelid swelling	0	0	0	0	0	0	0
Blepharospasm	Normal	Normal	Normal	Normal	Normal	Normal	Normal

**Table 3:** Grading of induced corneal ulcer severity at the beginning of treatment

**Parameters**	**Grade**			
	**0**	**1**	**2**	**3**
Discharge	None	Minimal	Moderate	Heavy
Conjunctival redness	None	Minimal	Moderate	Heavy
Size of corneal opacity	None	< 25%	25–75%	75–100%
Density of corneal opacity	Iris/pupil margin fully visible	Hazy	Cloudy	Iris/pupil margin not visible
Corneal surface	Smooth, epithelium present	Partial epithelial disruption	Epithelium absent	Descemetocele

**Table 4:** The data related to the culture/smear results (in 48 and 96 hours after ulcer induction) and corneal ulcer clinical score in the follow-up examinations (Days 2, 4, 6, 9, 12, and 15) in rabbits of the intervention (I) and control (C) groups

	**Culture/Smear**		**Corneal ulcer clinical score**					
	**48 hrs**	**96 hrs**	**Day 2**	**Day 4**	**Day 6**	**Day 9**	**Day 12**	**Day 15**
I1	+	–	3.75	4.00	4.00	0.00	0.00	0.00
I2	–	–	4.00	4.25	4.00	4.00	3.75	0.00
I3	+	+	7.75	7.75	7.50	4.00	3.75	1.25
I4	–	–	4.00	4.25	4.00	3.25	0.00	0.00
I5	+	–	7.75	7.50	3.75	3.50	0.00	0.00
I6	+	–	7.50	7.75	4.25	4.00	3.50	1.25
C1	+	+	4.50	7.25	7.50	8.00	8.25	8.75
C2	–	+	4.00	7.25	8.00	8.25	8.25	8.50
C3	–	–	3.75	4.00	7.50	8.00	8.25	8.00
C4	+	+	7.25	7.25	8.50	8.75	8.75	8.75
C5	–	–	3.50	3.75	7.25	8.00	8.25	8.00
C6	+	+	4.00	4.25	7.75	8.00	8.00	8.00
hrs, hours								

**Table 5:** Mean and standard deviation (SD) of corneal ulcer clinical score on different days during the follow-up period in both intervention and control groups

	**Time (day)**	**Control group**	**Intervention group**	***P*** **-value**
Corneal ulcer clinical score	2	4.33 ± 1.44	5.70 ± 1.97	< 0.001
	4	5.62 ± 1.78	5.91 ± 1.92	
	6	7.75 ± 0.44	4.58 ± 1.44	
	9	8.16 ± 0.30	3.20 ± 1.58	
	12	8.29 ± 0.24	1.83 ± 2.01	
	15	8.31 ± 0.27	0.41 ± 0.64	

**Figure 4:**
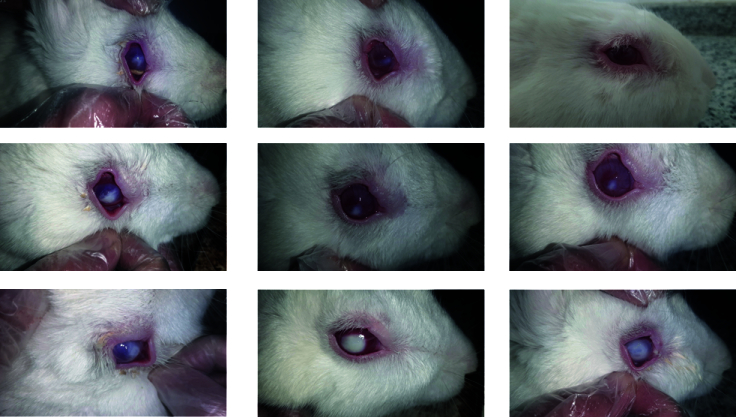
First row: Pictures of the right eye of a rabbit with therapeutic intervention on days 2, 9, and 12, from left to right, respectively, which shows that on the ninth day, the evidence of infiltration and the epithelial defect has completely disappeared. Second row: Clinical recovery process in a rabbit on days 2, 9, and 12, from left to right, respectively, with therapeutic intervention. On the 12^th^ day, the evidence of infiltration and the epithelial defect has completely disappeared, and corneal neovascularization is evident. Third row: Pictures of the right eye of a rabbit without therapeutic intervention on days 2, 9, and 12, from left to right, respectively, which show that the progress of corneal ulcer, along with the development of hypopyon and eventually corneal melting, is evident.

**Figure 5:**
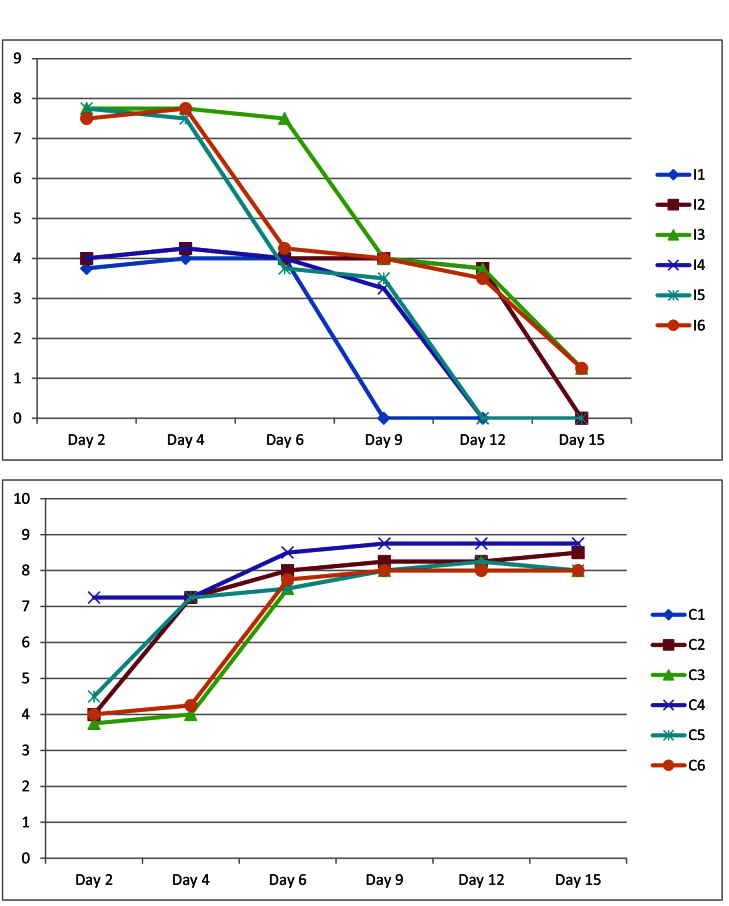
The course of changes in the severity of corneal ulcer based on the corneal ulcer clinical score in the intervention (I) and control (C) group rabbits in follow-up examinations on days 2, 4, 6, 9, 12, and 15.

**Figure 6:**
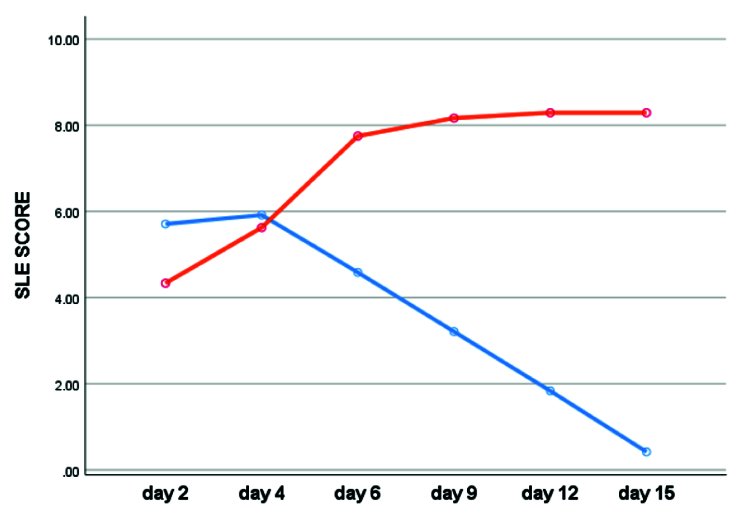
Comparison chart of the average clinical score of corneal ulcer severity in two intervention (blue) and control (orange) groups based on follow-up days.

### Clinical Findings of Corneal Ulcer Treatment

In this study, the severity of the ulcer in the rabbits' eyes was determined 48 hours post-treatment based on the ulcer grading table. Subsequently, clinical findings were assessed on the second, fourth, sixth, and twelfth days of examination based on the clinical classification criteria and then graded accordingly. The ceftazidime nanofiber, which had a 15% drug-to-polymer matrix and was produced by the Faculty of Pharmacy at Kermanshah University of Medical Sciences, released the drug at levels exceeding the minimum inhibitory concentration (MIC) of the drug for > 96 hours *in vitro* (details will be reported in another article). The *in vivo* validation suggested that the drug concentration was maintained above the MIC90 for *S. aureus* and *P. aeruginosa* for 96 and 120 hours, respectively.

The nanofiber's structure consisted of fibers measuring 150–250 nanometers in diameter, providing a slender and fine framework that enhances its compatibility with ocular tissue. Frequent examinations confirmed that the insert was secured in the lower conjunctival fornix (cul-de-sac) for three days. To ensure effective drug delivery, we replaced the insert every three days. Following the formation of the ulcer and prior to the initiation of drug treatment, a corneal sample was dispatched for culture and smear analysis to confirm infection with *P. aeruginosa* monopathogen. Additionally, corneal secretions were cultured 96 hours after the onset of the treatment to verify the effectiveness of the treatment based on corneal ulcer severity grading [Table 3]. After culture and smear samples were collected from the wound secretions, the results showed that four of the six treated rabbits tested positive for *P. aeruginosa* after 48 hours, while the remaining two rabbits tested negative. In the control group, three rabbits had positive cultures, while three had negative cultures. Moreover, 96 hours after the onset of the treatment and the insertion of the ceftazidime insert, cultures and smears of the wound secretions were collected again to assess the antimicrobial effects of the medicinal insert. The findings showed that three rabbits in the intervention group, which initially had positive cultures, tested negative in the subsequent examinations. However, one rabbit still had positive culture and smear results.

In this study, following ulcer induction, the severity of the rabbits' ocular condition was categorized based on a corneal ulcer scoring system. In the intervention group, one rabbit was classified as grade 3, three as grade 2, and two as grade 1. In the control group, two rabbits were classified as grade 2, one as grade 3, and three as grade 1.

During the treatment, out of the six treated rabbits, one that developed a mild ulcer showed no signs of an active wound or inflammation by the ninth day of examination. Two rabbits showed signs of subsiding inflammation and scar tissue formation by the 12 ${}^{\text{th}}$ day. In the remaining three rabbits, the examination on day 15 revealed evidence of scarring and the disappearance of active inflammation. During the examination, hypopyon formed in the anterior chamber of the eyes in three rabbits. This condition improved in two of the rabbits by day 6 and in the remaining rabbit by day 4 post-treatment. In the control group (six rabbits), which did not receive any therapeutic intervention, the severity of corneal involvement, such as the degree of infiltration, the level of hypopyon, and corneal melting, was evaluated at each examination. In each examination, these signs appeared more pronounced than in the previous assessments [Figure 4]. Table 4 and Figure 5 present the data related to the culture/smear results and corneal ulcer clinical scores for rabbits in the intervention and control groups in the follow-up examinations.

According to the results, the average clinical score of the corneal ulcers differed significantly between the two groups (*P* < 0.001) [Table 5; Figure 6]. This significant difference confirms the effectiveness of therapeutic intervention in corneal ulcers.

##  DISCUSSION

Corneal ulcers are among the leading causes of blindness worldwide.^[1]^ Their prognosis necessitates rapid diagnosis and therapeutic intervention, primarily through the use of topical eye drops. The primary challenge in drug delivery via eye drops is the limited volume that can be applied to the corneal surface due to its restricted surface area ( ∼ 10 μ L) and the removal of administered volume during the initial blink reflex, which is triggered by a sudden surge in tear production.^[6,7]^ Consequently, eye drops need to contain high concentrations of medication to counteract factors that contribute to low ocular bioavailability, including tears, blinking, lacrimal drainage, blood and lymphatic vessel flow in the conjunctiva, metabolic breakdown, and ocular and corneal blood barriers.^[6,7]^ Moreover, the high concentration of medications associated with bolus administration can lead to both local and systemic complications.^[20]^ There are also variations in drug concentration depending on the administration technique and patient compliance with the use of eye drops. Therefore, an efficient, noninvasive delivery system, such as nanofiber carriers, is required to control drug release and decrease dosing frequency.^[8,9]^ These carriers can deliver an appropriate dose of the drug to the site of action without preservatives, maintain an appropriate concentration of the drug throughout the day, and reduce the frequency of drug administration.

In this research, we successfully induced corneal ulcers in the rabbits' eyes. The pharmacodynamic evaluation of the nanodrug delivery system demonstrated the efficacy of the nanofiber insert in treating corneal ulcersinduced by* P. aeruginosa*. This study is the first to explore the effectiveness of a ceftazidime-loaded insert as an ocular drug delivery system.

In a study by Tanwar et al, a medicinal ofloxacin insert was prepared, and the drug release rate was examined both in laboratory and clinical conditions by placing the insert in the eyes of rabbits. According to their findings, the drug was released in the rabbits' eyes for over 24 hours and effectively prevented bacterial growth under laboratory conditions. However, it is essential to note that this study only examined the release of the drug in the eyes without evaluating infectious conditions.^[21]^ In a study by Taghe et al, ciprofloxacin was incorporated into the film and nanofibrous inserts. The film and nanofibers released the drug for 2 and 5 days *in vitro*, respectively, while the eye drop was effective for only 10 hours. Moreover, pharmacokinetic studies have shown that the nanofiber formulations exhibit 4.5–5 times higher AUC (area under the curve) in the eyes of rabbits than the eye drops.^[14]^

In 2021, Mirzaeei et al developed a 70–350 nm nanofiber insert that contained gentamicin and prednisolone along with appropriate mechanical properties and shapes. The best formulations showed a prolonged release of both drugs for 3–9 days and an antibacterial effect against *S. aureus*.^[22]^ Mirzaeei et al also developed ofloxacin nanofibers in 2021 that effectively retained drug concentration in the tear fluid of rabbits above the MIC90 for up to 95 hours.^[23]^

In 2016, Karataş et al conducted a study to examine the impact of formulation variables on the morphology of fibers and drug release in electrospun ofloxacin-loaded silicone fibers for ocular drug delivery. This study investigated the effects of several formulation variables on the drug release rate, including the ratio of polymer to drug, fiber size, diameter of electrospun fibers, thermal properties, and antibacterial activity. The findings revealed that the average fiber diameter decreased with a reduction in the amount of polymer in the initial composition. Conversely, the release of the drug was enhanced by increasing the amount of drug in the formulations.^[24]^

Moreover, in 2012, a study was conducted to investigate the use of vancomycin in novel electrospun sandwich-structured polylactide-polyglycolide/collagen nanofibrous membranes. The study was conducted *in vitro* and aimed to elucidate the drug release rates over 30 days. The findings revealed that these biodegradable nanofibrous membranes could release high concentrations of vancomycin, exceeding the MIC. Furthermore, the results demonstrated the effective activity of nanofibrous membranes. Overall, the authors suggested that using the electrospinning technique could produce biodegradable nanofibrous membranes suitable for the long-term delivery of various drugs.^[25]^

The main strength of this research is that it is the first to investigate the antibiotic effects of the nanofiber insert on induced corneal ulcers and clinical simulations. However, since the researchers did not intend to euthanize the animals, they relied on clinical examinations, tissue culture, and smear analysis instead of tissue pathology.

In summary, the ceftazidime-loaded nanofiber was significantly effective in treating corneal ulcers. As a result, this formulation could serve as an effective alternative to eye drops for treating corneal ulcers. It is important to note that while antimicrobial agents are the mainstay of treatment for ulcers, other medicinal compounds such as collagenase inhibitors, topical corticosteroids, and anti-inflammatory agents are also utilized.^[26]^ However, to avoid any potential drug interference, we did not use these categories of drugs in this study. Future studies are recommended to consider the effects of these drugs. While laboratory evidence supports the effective release of the drug for > 72 hours in animal studies, further human research is recommended to confirm these findings.

##  Financial Support and Sponsorship

The authors acknowledge the financial support from the Research Council of Kermanshah University of Medical Sciences for this work (Grant number: 50003749).

##  Conflicts of Interest

None.
